# Academically Produced Air Pollution Sensors for Personal Exposure Assessment: The Canarin Project

**DOI:** 10.3390/s21051876

**Published:** 2021-03-08

**Authors:** Boris Dessimond, Isabella Annesi-Maesano, Jean-Louis Pepin, Salim Srairi, Giovanni Pau

**Affiliations:** 1 Computer Science Laboratory, University of Pierre et Marie Curie, LIP6, NPA Team, 4 Place Jussieu, 75005 Paris, France; giovanni.pau@unibo.it; 2 EPAR Team, Medecine Faculty of Saint-Antoine, IPLESP, INSERM & Sorbonne University, 27 rue Chaligny, 75571 Paris, France; isabella.annesi-maesano@inserm.fr; 3 Laboratoire HP2, Université Grenoble Alpes, Grenoble, INSERM, U1042 and CHU de 24 Grenoble, France; JPepin@chu-grenoble.fr; 4 Centre for Expertise and Engineering on Risks, Urban and Country Planning, Environment and Mobility—Ile de France Territorial Division, Mobility Department, 12, rue Teisserenc de Bort, 78190 Trappes-en-Yvelines, France; salim.srairi@cerema.fr

**Keywords:** Internet of things, sensor mesh architecture, particulate matter sensor, indoor air pollution, exposure assessment, health impact, mobility, sleep apnea

## Abstract

The World Health Organization has estimated that air pollution is a major threat to health, causing approximately nine million premature deaths every year. Each individual has, over their lifetime, a unique exposure to air pollution through their habits, working and living conditions. Medical research requires dedicated tools to assess and understand individual exposure to air pollution in view of investigating its health effects. This paper presents portable sensors produced by the Canarin Project that provides accessible, real time personal exposure data to particulate matter. Our primary results demonstrate the use of portable sensors for the assessment of personal exposure to the different micro-environments attended by individuals, and for inspecting the short-term effects of air pollution through the example of sleep apnea. These findings underscore the necessity of obtaining contextual data in determining environmental exposure and give perspectives for the future of air pollution sensors dedicated to medical research.

## 1. Introduction

Modern societal development relies heavily on the consumption of fossil fuels that continually release more harmful gases and particles in the atmosphere, negatively impacting both the climate and human health [1]. Air pollution is a major cause of disease and premature death, and is the single largest environmental health risk in Europe [2].

In an urbanized environment, individuals are exposed to outdoor air pollution created by industry, traffic and domestic heating. However, individuals spend most of their lifetime indoors in settings where they are exposed to a different set of sources of air pollution, including combustion for heating and cooking, building materials, cleaning products, or cigarette smoke. As a consequence, exposure at the personal level comprises a unique composition of air pollutants as the individual moves across different environments. It is, thus, important when investigating the health impacts of air pollution and involved mechanisms to prioritize personal exposures over ambient concentration values.

Air pollution has historically been measured using fixed air pollution stations, providing high quality data at one geographical coordinate. The paradigm of air quality monitoring has changed as low cost networks of air pollution monitors are able to provide a higher spatio-temporal resolution than the more expensive reference stations [3,4,5,6,7,8,9]. Additionally, satellite imagery, although limited by its resolution, allows for the assessment of air pollution where other means are not available. Collectively, these measurements are largely used in assessing health effects of air pollution at the population level. However, they cannot provide individual level data, nor can they provide assessments of indoor air pollution, of living and working conditions, behavior and habits, ventilation, cooking habits or the use of chemicals for cleaning, which will considerably impact the quality of the air for a given person. Thus, individual level data enable medical researchers to more accurately depict and understand how air pollution impacts human health [9].

The rise of wearable smartwatches, with fitness and sleep activity trackers, has created a cultural phenomenon called the quantified self, whereby members of the general population voluntarily wear tracking devices that continuously log their data, in exchange of potential improvements in their quality of life or physical performance. Real-time monitoring of air pollution levels can alert subjects about sudden peaks or slow rise of air pollutants, enabling them to change their behavior (in case of indoor air pollution) or to avoid the pollution source (in case of outdoor air pollution). Public adoption of wearable air pollution sensors and health monitoring devices has considerably increased over the last few years.

Sensor is a broad term that describes both the technology sensing and communicating physical change to other electronics, as well as the whole device embedding that technology and the necessary hardware to be used as a standalone device. A wearable sensor is composed of an enclosure, embedded battery, one or multiple sensors, integrated circuits, passive electronics and a microcontroller unit (MCU) running a dedicated software and possibly remote server hosting web services to store and subsequently access the data. Technology in sensing modules is evolving at a fast pace, as many companies are developing and producing small, efficient, affordable and reliable sensor modules, enabling the end products, the wearable sensors, to produce a level of quantity and quality of data that has never been reached before.

In the field of air quality sensors, commercial devices and original equipment manufacturer (OEM) low-cost sensors have been benchmarked by the scientific community [10,11,12,13,14,15,16,17,18,19,20] and multiple methodologies and tools have been developed for validation [21,22,23,24]. Such sensors have been considered for crowdsensing applications [25,26,27,28,29,30,31]. However, there are fewer wearable sensors produced by research projects [32,33,34,35,36,37], and the amount of commercial wearable sensors aimed at the general public, rather than use in industrial settings, is surprisingly even more limited [38,39]. While wearable and/or portable air pollution sensors do exist, accessing real-time geolocalized data obtained by these sensors is not necessarily available to third parties, such as a research team. Thus, Canarins were created with this purpose in mind—to make available a robust, validated, portable sensor for public health research requiring continual individual exposure information.

The primary purpose of this paper is to present the application of the Canarin portable sensors for medical research. Specific objectives include the description of the Canarin as a tool for assessing individual exposure to particulate matter (PM) in real time in relation to mobility (as in the POLLUSCOPE [40] project) and to associate it with sleep apnea (as in the POLLAR project). The Canarin project has already leveraged today’s best available technologies to produce granular level data. Through our work on these projects, we have determined that further efforts in contextualizing the acquired data must be made. The final goal of this paper is to describe an innovative approach in sensor architecture design, specifically aimed at improving the interpretation of personal exposure to air pollution.

## 2. Materials and Methods

### 2.1. Overview of the Canarin Project

The Canarin project unites an international team of researchers and students with a common goal: to produce and utilize low-cost air pollution sensors to assess individual exposure to air pollution and its health effects. While alternative sensors are commercially available, they often are required to connect to an external device for data transmission, such as a mobile phone, and data are often shared only with the user, rather than a third party. As such, this project aims to solve issues such as connectivity, cost, size, data acquisition and ease of use for medical and health study participants.

Canarin sensors use commercially available development boards and modules, which have the advantage of having been already implemented and tested [41]. We selected an optical particle counter (OPC), and made efforts to integrate modules in an enclosure following the manufacturer recommendations, ensuring the best expectable performance. The rest necessary components are mounted on printed circuit boards (PCBs) that we manufactured externally. No post-processing is applied to the data before transmission.

The first Canarin was created in 2016 by the Asian Institute of Technology, Sorbonne University, and the University of Bologna, who partnered in the frame of the SEA-HAZEMON project [42]. A series of Canarins were deployed, acting as low-cost fixed air pollution monitoring stations, collecting data on Particulate Matter (PM), namely particles of 1, 2.5 and 10 µm of diameter (PM_1_, PM_2.5_, PM_10_), from the recurring haze events in the Southeast Asia region. Using portable sensors such as these to probe human exposure had been previously investigated at the Asian Institute of Technology [43]. In 2018, the Canarin II portable sensors were upgraded to include multiple wireless connectivity options and a GPS (global positioning system). [44] They were then dispersed over the urban environment of Bologna, while mounted on publicly available e-bikes. The ability to visualize geolocalized air pollution data in real time was a major improvement. Concurrently, other Canarin II devices were benchmarked by the POLLUSCOPE project, which required both mobile and affordable solutions to assess individual exposure to air pollution in the Île-de-France region. When tested against particulate matter sensors commercially available at the time, the Canarin II performed the best, as reported by national experts [21]. It was, therefore, selected and used extensively by the POLLUSCOPE project. Several Canarin II have also been deployed in the Johannine Library of Coimbra to monitor the impact of PM on its ecosystem, where the bat population has an essential role in the preservation of the library by eating the bookworms during the night [12]. These Canarin II are still acquiring data continuously to this date. In 2019, a smaller and lighter Canarin was created; the Canarin Nano. It has been used in the POLLAR project, with the primary goal of exploring whether sleep apnea is aggravated by exposure to microparticles [45].

### 2.2. Canarin Nano

The hardware of the Canarin II runs on Linux, and therefore, provides a good amount of processing power and software development possibilities. The main board consumes approximately 3 watts, requiring a battery able to sustain a normal day of use. The size and weight of the device (around 1.2 kg) is not ideal for individuals who have to carry the device all day long, especially if they are affected by respiratory diseases, as was the case in the POLLUSCOPE project. Such drawbacks had been previously outlined during the performed benchmarking [21], and volunteers of the POLLUSCOPE project commented on these aspects. Technological advancements in microcontrollers, PM and gas sensing technologies have made size reduction possible without compromising the quality of the acquired data. The Canarin nano uses a microcontroller based on the ARM Cortex-M processor instead of the more power hungry UDOO NEO FULL [46] Cortex-A9 processor used on the Canarin II. Onboard, a 3G modem, flash memory, 4000 milliampere hour (mAh) battery, a GPS module, a particulate matter sensor (PM_1_, PM_2.5_, PM_10_), a temperature humidity and pressure sensor all manage to provide the same level of performance previously offered by the Canarin II, while staying on for up to 14 h on a single charge. Improvements in particle sensor design allowed for a slimmer device, and recent development in electrochemical sensors have allowed, for the first time, to accommodate a volatile organic compound (VOC) sensor. The VOC sensor is mounted on a secondary printed circuit board (PCB) header board that can be easily plugged in and out of the main board. This secondary PCB header board opens the possibility to upgrade to newer electrochemical or metal-oxide gas sensors, without needing to remanufacture the entire device. Flash memory has been used to prevent data loss if the on-board 3G modem loses connectivity with cell towers. A third party IoT (Internet of things) or M2M (Machine to machine) SIM (subscriber identity module) card provider has been selected instead of the default Particle cloud services [47]. Table 1 provides some of the components used in the Canarin Nano.

The cloud infrastructure server is hosted by Amazon Web Services (AWS) through an EC2 T2 instance [48]. One vCPU (virtual central processing unit) and 1 GB of RAM (random access memory) are enough to provide service for approximately 100 Canarin Nanos. Multiple docker containers [49] run Python scripts to ingest data. The database is also hosted by AWS in Frankfurt, following the GDPR (General Data Protection Regulation). The front end [50] allows users to download or visualize data in real time.

**Figure 1:**
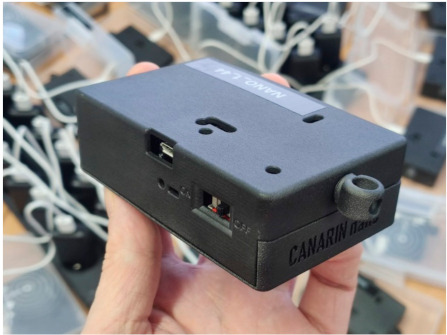
A Canarin Nano. The hook in the foreground is for carrying the device.

Once the first prototypes provided satisfying performance in a real-world scenario, a redesign of the device occurred, to switch from locally soldered and printed materials, to a custom PCB and a fused deposition modeling (FDM) 3D printed enclosure, produced externally in larger quantities. Complexity to source the bill of materials, logistics and delays increased the overall cost per unit. Any possibility to streamline the production chain was abandoned due to lack of manpower. Redesigning the entire device enclosure and PCB successfully eased the assembly and maintenance of the sensor, while greatly improving reliability. The enclosure provides protection for the electronics, visibility for multiple light-emitting diodes (LEDs) and accessibility to the available ports, buttons and switches, see Figure 1. The Canarin Nano is designed to be always on, as the instrumented patients and volunteers should have no interactions with the device other than to carry it with them. The participants can, however, choose switch it off, disabling the on-board GPS module, when privacy concerns are involved.

Having a sensor permanently connected through 3G provides several advantages; sampled data is sent each minute to our cloud service, and is immediately available for the researcher to visualize or download. The patients and volunteers can, therefore, be monitored multiples times per day, limiting the risk of data loss. Setting changes or full firmware updates can be triggered over-the-air, without the end user even noticing it happening. Reducing the sensor size and weight helped create a device able to fit more of the medical researchers’ requirements than other commercial sensors in the sub-thousand euros price range. Improvements will occur as student and researcher creativity will continue to drive the Canarin project.

### 2.3. POLLUSCOPE Project

The primary goal of the POLLUSCOPE project is to create a participatory observatory for measuring air pollution at the individual level with low-cost portable sensors. One of the first tasks of the project was to test, compare and identify which sensors will be fit to the task. Selected sensors have been evaluated by a panel of experts, who compared these sensors against reference instruments [21] to ensure that the performance of the selected modules was satisfactory enough to be used in real-life mobility scenarios. The Canarin II went through both static and dynamic tests and was selected as the best particulate matter (PM) sensor. The project has since been using the Canarin II. POLLUSCOPE is a long-term project, and sensors can spend months unused. To moderate the risk of having a faulty sensor before going through several weeks of measurements, all selected air pollution sensors were regularly handed over to the project’s air pollution experts to be tested against to reference instruments. For the particulate matter, a Thermo Scientific^TM^ 1405-F tapered element oscillating microbalance (TEOM), for PM_10_, and a ThermoFisher TEOM 1400 were used [21]. If sensors displayed unexpected behaviors, they were set aside for servicing.

**Figure 2:**
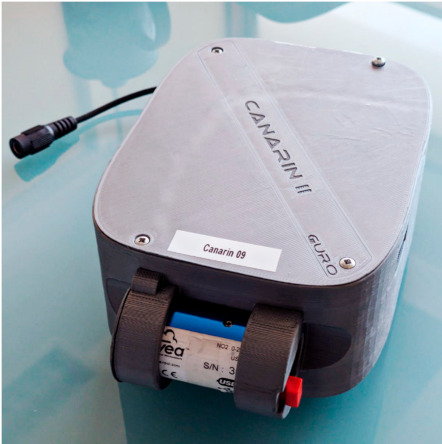
Canarin II in grey. The Cairsens NO_2_ is conveniently attached to it.

The second goal of the project was to assess individual exposure to air pollution as a function of mobility. Three population groups were considered: volunteers from Versailles Grand Parc (VGP), participants of the population-based survey RECORD [51] and volunteers affected by respiratory diseases. VGP volunteers had to fill an online survey, and provide written consent for their data to be used. Volunteers were required to wear POLLUSCOPE’s sensors, including a Canarin II (Figure 2), for one week. The volunteers were asked to keep the sensors turned on at all times, and to let them charge when possible. The minimum sensor set is composed of a Canarin II (laser diffraction particulate matter sensor), a portable electrochemical NO_2_ (nitrogen dioxide) sensor attached to it (Cairsens NO_2_ [52]) and a tablet (Archos 101 Oxygen 4G) to manually log activity through a dedicated app and provide a Wi-Fi access point for the Canarin II.

Each air pollution sensor used by the project samples air pollution every minute. Depending on the cohort, the sensor set can be completed with an AE51 (optical black carbon (BC) sensor, aethalometer) and a Bluetooth low energy (BLE) connected portable spirometer, as well as an oximeter to log health parameters, three time a day. Volunteers were invited to fill an online survey at the beginning of the study in order to collect some important socio-economic data.

The third goal of the project was to provide the participants with valuable feedback on their exposure to air pollution. Automating such a task was only limited by our ability to understand the available data. Relevant information can be provided if we know in which context air pollution exposure happens. We demonstrated that the multiplicity of portable sensor’s data, completed by user’s annotations, can be used to improve the knowledge on personal exposure to air pollution.

### 2.4. POLLAR Study

**Figure 3:**
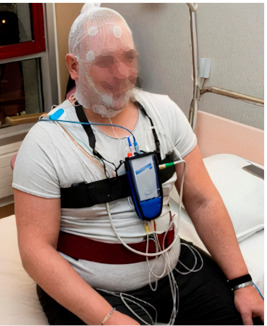
Subject instrumented before polysomnography (PSG) screening at the hospital.

The EIT-HEALTH POLLAR clinical study [45] began in March 2019, with the objective of estimating the impact of air pollution and pollen exposure on rhinitis, asthma and sleep. Pollen data were obtained through the Réseau National de Surveillance Aérobiologique (R.N.S.A., French aerobiology network [53]). The impact on sleep was studied at the Grenoble Alpes University Hospital where sleep apnea patients were recruited to perform, at two different periods, a polysomnography (PSG), followed by three days of air pollution exposure using a Canarin Nano or a Canarin II. A PSG is a comprehensive sleep study that requires the subject to stay at the hospital for one night equipped with monitoring devices (Figure 3). The main PSG outcome considered in the present study is the severity of the sleep apnea, given by the apnea-hypopnea index (AHI). The Canarin II and Canarin Nano sensors allowed for the first time the comparison of PSG results with granular air pollution data obtained both during and after sleep.

### 2.5. Quantifying Individual PM Exposure Relative to Mobility and Activities

Individual exposure to PM was expressed as a concentration in µg/m^3^. Mobility data were acquired through the Canarin II and tablet onboard GPS. In the results are displayed the spatial tracing of the PM_10_ data acquired by one volunteer, a member of the POLLUSCOPE consortium, totaling six and a half days of data (156 h) from two sampling sessions in November 2019. This volunteer was selected as he is a collaborator working at the CEREMA Île-de-France and have agreed to publish his data. Both raw GPS and Canarin II data were used.

### 2.6. Statistical Methods

We used Python’s NumPy and Pandas libraries to produce the results provided in the tables. The Canarin data were downloaded from the POLLUSCOPE projects data hosting server. POLLAR’s Canarin data were obtained from the Canarin project website [50], through a password-protected web page. Data produced by the polysomnography and Canarin deployment planning of the POLLAR study were provided through an Excel file. No post processing was applied to compensate for data loss or user errors; all figures and tables were produced using raw unprocessed data. Matplotlib and Seaborn were used to create the figures. Mobility data figures were produced using QGIS and an Open Street Map layer for understandability. In the study of mobility, the notion of points of interest (P.O.I.) introduces two contrasting clusters with regard to individual exposure and device behavior.

## 3. Results

### 3.1. Individual PM Exposure as a Function of Mobility (The POLLUSCOPE Project)

The data presented below were acquired by the collaborator working at the CEREMA^3^. The data are composed of two sampling periods of 8 days each (384 h in total). Table 2 shows, for each of the sampling periods, the average air pollution levels in µg/m^3^ and the count of the total number of recorded samples. All sensors sampled air pollution once per minute. Over the course of each 8-day period, measurements were taken 6040 (period A) and 4227 (period B) times, representing approximately half and just under half of the potential maximum of 11,520 measurements, respectively (Supplementary S1).

**Figure 4:**
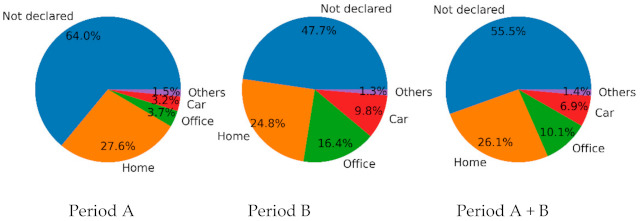
Distribution of the activities per period.

The volunteer was exposed to higher concentrations of air pollution during the second period (Table 2 Period . Activity data distribution in Figure 4 shows we lack activity for 55% of the acquired samples (Figure 4 “Period A + B”). Available activity data may contain mistakes and require post-processing. A higher percentage of activity data has been obtained from the user for the activities “Car” and “Office” (Figure 4 “Period B”). Any difference observed in terms of exposure to air pollution between the two periods could be explained by the lack of data, or by the over representation of specific activities. This underscore that sensors should ideally be never stopped during data logging, and how annotations that contextualize the sensor’s data are mandatory to provide a trustworthy analysis.

**Figure 5:**
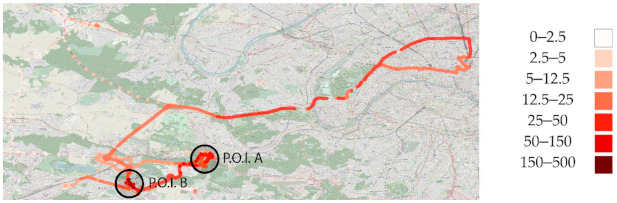
Spatial tracing of PM_10_ (µg/m^3^) for both periods A and B.

Figure 5 shows the two points of interest, circled in black, that we are going to focus on. The rest of the map displays the entirety of the spatial movement recorded by the GPS over the two periods. In the north-west, a single ride away from an urban area is visible, translated into lightly colored low PM_10_ exposure. PM_10_ exposure is higher along the Paris’s ring road (“Boulevard Périphérique,” in the north-east corner), and decreases away from the main road.

**Figure 6:**
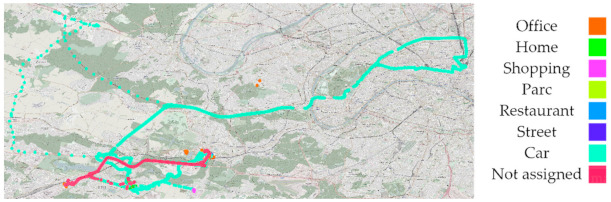
Spatial tracing of activity annotations for both periods A and B.

In Figure 6, in the south, a commute to a store is marked by a bright violet end and a noticeably higher PM_10_ exposure (see Figure 5). Both P.O.I. in Figure 5 contain a cluster of samples, which generally indicate a working or living place. A pink trace in Figure 6 shows a commute by car or public transports that have not been manually logged by the volunteer; therefore, the recorded PM data are not traced in Figure 5.

**Figure 7:**
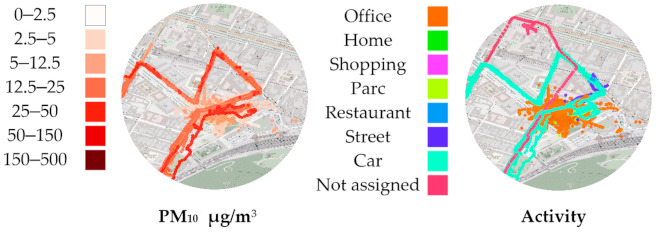
Geographical point of interest A for particulate matter (PM_10_) measurements “Workplace” cluster surrounded of other activities.

Zooming in on P.O.I. A confirms that we are observing the volunteer’s annotated “Workplace” (Figure 7). The missing tracking activity information also lacks PM_10_ samples. The “Workplace” cluster of points shows a very common case of raw GPS data noise caused by less-than-ideal GPS signal reception. The accuracy of localization drops indoors. From this figure, we can observe how lower levels of PM_10_ have been recorded indoors compared to the surrounding urban areas. The cluster in the west in Figure 5 and Figure 6 correspond to the volunteer’s secondary workplace, in a less urbanized area, with lower PM_10_ levels.

**Figure 8:**
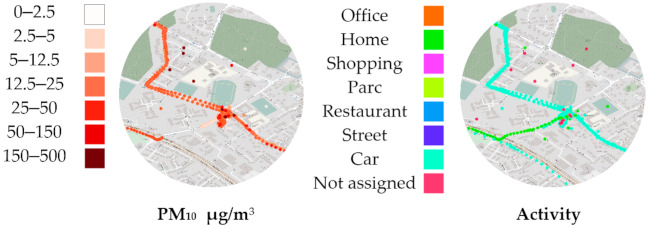
Point of interest for particulate matter (PM_10_) B “Home” cluster with lack of GPS reception.

P.O.I. B (Figure 8) shows some randomly localized samples with high PM_10_ values. These few points around our P.O.I. comprise 6905 PM_10_ samples, the higher samples being displayed first. This indicates an indoor location where GPS reception was not possible, and samples are all displayed on the last found location. Notice how multiple commutes by car leads to this cluster, with one accidentally marked as “Home” instead of “Car,” in green. This mistake will negatively impact the estimated exposure to air pollution at “Home.”

**Figure 9:**
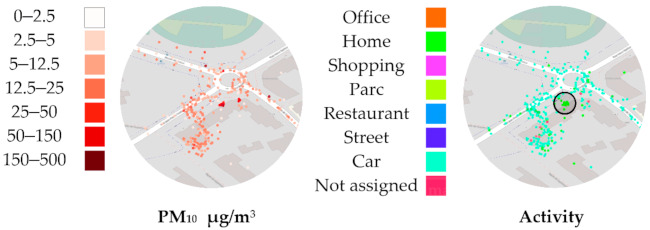
Point of interest for particulate matter (PM_10_) B, close up of “Home.”

Zooming further into the cluster (Figure 9) reveals the volunteer’s “Home.” Below the center, among the noise, we can guess where the car is supposedly parked. Circled are a total of 321 PM_10_ samples, which are heavily concentrated on pinpointed coordinates, indicating what could be the real “Home” location, possibly next to a window. However, we confirmed with the volunteer that no GPS samples are located precisely on the “Home” location. Without more contextual information, the lack of indoor GPS reception and the low count of heavily concentrated samples could mislead algorithms aimed at filling or correcting activity data, or bias the estimated PM_10_ value at “Home,” obtained from GPS data clustering.

Table 3 provides the average air pollution levels per activity, according to user’s annotations. PM_10_ levels in “Office” are low, as observed previously in Figure 7. “Home” has the highest PM exposure averages, and “Car” is the activity with the highest overall air pollution exposure, specifically regarding NO_2_ and BC levels. The average PM_10_ value in the circled region in Figure 9 is 34.1 µg/m^3^, double what was obtained using the annotated data. NO_2_ and BC levels during the activities “Street” and “Car” are predictably higher than any other activity.

### 3.2. Individual PM Exposure in Relation to Sleep Apnea (The POLLAR Study)

**Figure 10:**
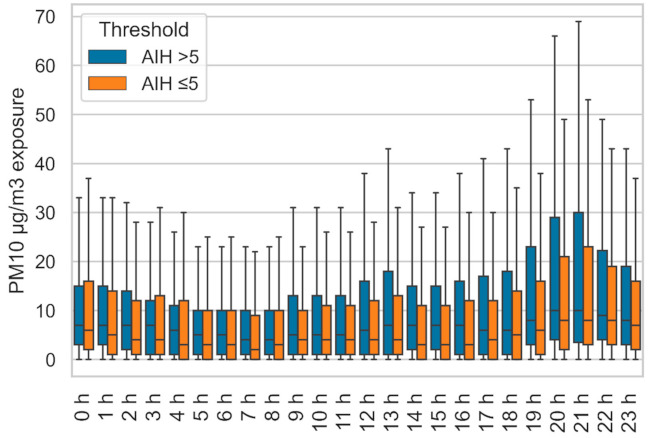
Hourly PM_10_ concentrations according to the apnea-hypopnea index (AHI) group.

Figure 10 displays the hourly average PM exposure for POLLAR study subjects affected by sleep apnea (21 subjects with average AHI > 5, in blue), and subjects with normal AHI (22 subjects with average AHI ≤ 5, in orange). In each group, the upper value of the box plots indicates how much some subjects were exposed to unusually high values of PM_10_. In this figure, the extreme outliers were hidden, as one volunteer was consistently recording excessively high values of PM overnight, with PM_10_ samples above 1000 µg/m, which exceeds the functioning range of the PM_10_ module. Such values can be recorded by OPCs when exposed to e-cigarettes vapor, but no other information is available to contextualize the data. The median values are very close between the two groups. Between 9 a.m. and 9 p.m., the 75th percentile PM_10_ values were higher in the group affected by sleep apnea.

## 4. Discussion

### 4.1. Quantification of Individual PM Exposure

We have shown how portable air pollution sensor data can be interpreted based on user’s information about their activity. We also saw how a scrupulous data review could partly correct activity data in case the user neglected to log their activity data. Data completion and activity correction algorithms are under development within the POLLUSCOPE project.

When assessing personal exposure, volunteers are required to be constantly aware of their obligation to keep the devices charged, to remember to carry the devices and possibly also track their daily activities. This requires them to perform multiple manual operations during the busiest moments of their day. A mobile app is delivered on a tablet, and is available on Android phones for the user’s convenience. The app has been developed by the POLLUSCOPE project to ease this annotation step, which was historically done on paper notebooks. Volunteers are also asked to log in possible causes of air pollution level changes: close proximity to any pollution sources, ventilation from opening a window, proximity to cooking or smoking or other micro-environment changes. We observed how the end user could easily be a source of error and oversight or worse, abandon their duties mid-way through the measurement period. Portable air pollution sensors produce detailed data, but key information about the context is necessary to understand in detail the underlying causes of pollution change within the environments and the effects they may have on health outcomes.

### 4.2. Individual PM Exposure in Relation to Sleep Apnea

POLLAR successfully showed, as a pilot study, how personal sensors can open new opportunities for public health research. The study faced technical difficulties during the first months; prototypes of the Canarin Nano were not equipped with flash storage, leading to some data loss when no 3G coverage was available. Keeping a locally stored, continually updated database in addition to the cloud service would be the optimal option, but was not technically possible at that time due to the hardware limitations of the prototyping board selected. Legal authorization to record geolocalized data was not obtained; therefore, GPS had to be remotely disabled. The study design to assess the personal exposition to air pollution during the days following the PSG screening made it impossible to investigate if pollution exposure days prior to the screening leads to a degradation of the sleep quality. Comparing the two periods using a linear regression gives no correlation between the averaged PM exposure and the AHI. Such analysis might produce interesting results if exposure data were recorded anterior to the PSG screening.

### 4.3. Suggestion of Sensor Architecture Design for Academic Research

We saw how relevant information could be obtained directly from the user; inside the POLLUSCOPE project, we asked our volunteers, prior to delivering a set of sensors, to actively log their activity or pollution events during the following week of data acquisition. For this, however, we relied only on the users’ awareness and good will. We are still missing crucial information, as the POLLUSCOPE participants were never aware when the sensors detected a drop or increase in pollutant concentration values. Switching from requiring the user to manually annotate events to automatically prompt the user for annotations, based on the sensor’s live measurements, would improve considerably the quality and quantity of the available annotated data. In the paragraphs bellow, we will be detailing how a sensor mesh aimed to automate the detection of these events could be the key to expand the scientific knowledge on personal exposure to air pollution, as it would dramatically increase our capacity to analyze more precisely the granular data produced by these modern portable sensors.

**Figure 11:**
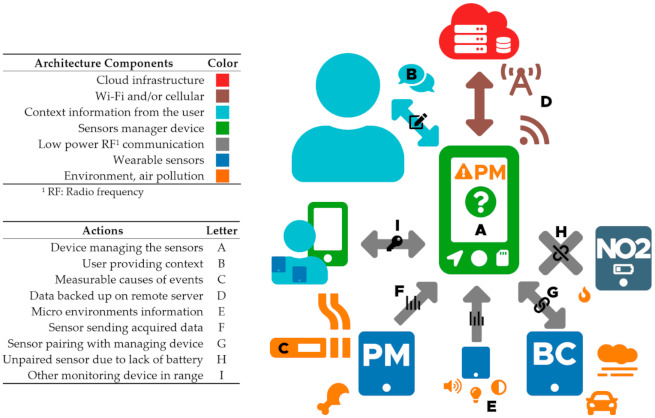
Illustration of the air pollution sensors mesh architecture.

To address this issue, we are suggesting a new approach in the development of air pollution sensors; through the creation of a sensor managing device at the center of a sensor mesh network (A). The goal of this managing device is to obtain live information from both the sensors and the user. The managing device would act as the primary human–machine interface for all the other sensors, displaying the acquired sensor data, remaining battery life and other necessary information in real time. If an air pollution event is detected, the managing device will notify the user and prompt them to provide necessary information to contextualize for the event (B). Users’ smartphones could be used to interface with the managing device. However, the managing device should keep its full ability to communicate with the user and register its inputs even if the user’s smartphone is turned off (Figure 11).

The managing device should be able to react within seconds, reaching user for feedback while it can still be aware of the causes of the event (C). Filtering has to be scaled for each sensor’s characteristic to avoid sending false or successive notifications. The managing device should be built around a powerful enough MCU such as PJRC’s Teensy boards [54] or development boards based off STMicroelectronics STM32H7 series [55]. Both score above 2000 in CoreMark [56], a microcontroller-dedicated benchmark. A good candidate for our application would be the Portenta H7 [57] from Arduino. It is equipped with a MIPI [58] interface, allowing the integration of high quality touch screens, has wireless connectivity with on-board Wi-Fi and Bluetooth modules, as well as an integrated battery charger for lithium polymer (Li-Po) batteries. These developing boards would provide enough processing power to display a graphic user interface (such as LVGL [59]), while performing continuous processing of both the live acquired data and previous user records. Cellular network modules, low power RF (radio frequency) modules (such as nRF24L01 [60]) and other desired modules and components would need to be interfaced through a custom PCB.

The monitoring device should aim to eliminate any need of post-processing the annotated context data. We were able to experiment within these projects the ways in which deploying sensors in the field can be a burden, each sensor having its own requirements. To solve this issue, the managing device would streamline the entire process, as individual sensors would be paired and start displaying data and battery levels within seconds, on one unique device.

By using a dedicated device instead of a smartphone or tablet, we solve any concerns of hardware limitation (e.g., RF communications with sensors), uniformity of hardware (embedded movement and sound sensors, GPS signal consistency) and software limitations (operating system versions, privacy safeguards and battery optimization limiting constant monitoring of the sensors). Commercial portable sensors, in the best scenarios, are connected to the subject’s smartphone via Bluetooth Low Energy (BLE), and use a proprietary app. This reserves the battery life necessary to communicate with a server and still allows the user to visualize sensor data in real time on their smartphones. It becomes cumbersome, however, for studies requiring subjects to wear multiple sensors; the subjects then have to go through multiple apps. Unless a standard emerges from the industry, we do think that a dedicated device would iron out these issues.

In case of connectivity loss with the remote server, all data would be preserved locally, and the architecture would continue functioning as expected. The possibility of connectivity loss by itself justifies the need of running event detection locally on the managing device. Both the sensors and monitoring devices should be able to withstand daily use and keep functioning independently of their orientation, weather conditions and the user’s activity (aside from swimming). Remote monitoring of individual sensors should be possible from a web service. Alerts could be sent to users or technicians if a dysfunction is detected. All logged data would be backed up locally and stored permanently on the remote server. Data should be both accessible directly from the monitoring device as well as on the web service (D). A reinitialization clearing the monitoring device’s internal database should be performed by a technician for privacy concerns prior to delivering it to another user.

Existing pollution sensors are dedicated devices, rarely monitoring parameters non-related to air pollution. As we would naturally use GPS to sense an environment change, we could, in addition, use ambient color temperature to sense indoor fluorescent bulbs or outdoor natural light. We are, therefore, suggesting to leverage the live analysis done locally by the managing device, by sensing other parameters using affordable and already available hardware. Light intensity, color temperature, sound spectrum and motion sensors would provide useful information to identify micro environments [35] (E). These sensors could be integrated with the monitoring device, or delivered as an optional sensor to pair with the monitoring device.

Power consumption is one of the main factors when considering designing new hardware [61] (p. 297). In the case of mobile sensors, increasing the battery life is always at the cost of the weight and size of the battery. Current meters for low-powered circuits have recently been made affordable [62,63]. Paired with a digital oscilloscope, such tools can read currents with values as low as nanoamperes. During the hardware and software development, the power consumption of integrated circuits and module should be monitored and optimized. Tasks such as geolocation, internet connectivity and storage should only be done by the monitoring device. Sensors that are paired to the monitoring device should not be designed to work as a standalone device. They should instead be designed to conserve battery life and communicate only with the managing device.

They should pair with the push of a button and start sending data to the monitoring device immediately. All sensors communicate to the managing device through the same low-power short-range radio communication (F), enabling ultra-fast and energy efficient data exchange between the sensors and the managing device. Between any data exchange, the microcontrollers running the sensors can be sleeping, saving on battery consumption. Because these communications are not secure, no sensitive information is transmitted by the sensors through low power RF; only the sensor’s unique identifier, its model type and raw data. Transmission with the sensors is bidirectional only during pairing (G). If a sensor paired is lost (H), the sensor will be turned off and the user will be notified. Monitoring devices should be able to advertise to other monitoring devices which sensors they are paired with, ensuring that no other monitoring devices in range could accidentally pair to these sensors (I). For sensor testing and calibration, to alternatively use sensors without a monitoring device, a much simpler receiving device without advanced processing or connectivity capabilities could be proposed.

Users should not have to proceed to any kind of manipulation of the sensors other than charging them. Sensors should have a uniform charging port. All interactions with the managing device should be easy enough to be performed by someone with no knowledge of the devices. The managing device should be smart enough to notify the user if a location’s samples are unusually high or low, prompting them to provide information accordingly, possibly triggering user behavior, and then prompting them again to document the event if another change in air quality is observed.

We acknowledge that the monitoring device requires considerable efforts in software and hardware development. The developers should ensure that the live data analysis done on the monitoring device is relevant for both the user and the data scientist. On the other hand, we would eliminate the need for standalone sensors, reducing for each unit of sensor; required hardware (cost, weight, size), software and electronic development time, manufacturing and servicing costs. Prototyping modules (low power RF transmitters, Li-Po battery chargers, voltage regulators) are very affordable and already available. Custom PCBs can be rapidly designed to interface modules, sensor modules and other essential hardware together. All in all, this approach would enable rapid integration and real-world testing of the latest air pollution sensing modules as they hit the market [12]. The concept we just described goes beyond solving the burden of the lack of contextual data. Users would be continuously informed on the quality of the air they breathe, and the effects of their behavior. We, therefore, expect to also drastically reduce the accidental data loss that usually occurs, by increasing the user’s dedication and awareness.

## 5. Conclusions

In this paper, we presented the Canarin project, and two academically produced sensors: the Canarin II and the Canarin Nano. These sensors were tested against other similar sensors and compared to reference monitors, proving their performance capabilities. As these sensors were designed to aid in health studies, they were trialed in two projects, POLLUSCOPE and POLLAR, where we investigated their utility and ease of use with real cohorts of study participants. With a POLLUSCOPE volunteer, we demonstrated the advantage of applying contextual activity data to the pollutant concentration level data in order to understand personal exposure and what that means on a daily basis. Collectively, these efforts highlight how the lack of contextual information is one of the shortcomings faced when acquiring granular data. We addressed this issue by offering the concept of an air pollution sensor mesh that would considerably improve the knowledge on individual exposure to air pollution by soliciting the user to provide information based on a sensor’s input.

Because the acquired knowledge and experience is invaluable, we are advocating for researchers and students from heterogeneous fields to partner together and create their own devices, as has been done for the Canarin project. Finally, the scientific community should seize the opportunity to create its own open standard for portable sensors, using the best hardware for the task, with an architecture focused on ease of use, subject’s engagement and, indeed, data contextualization.

## Figures and Tables

**Table 1:** Canarin Nano components.

Components	Reference
Development board	Particle Electron 3G-U270
Microcontroller	STMicroelectronics STM32F205
GSM ^1^ module	U-BLOX SARA-U270
Cellular antenna	Molex Part Number 2072350100
IoT ^6^/M2M ^7^ SIM ^8^ card provider	ThingsMobile
GPS ^2^ module	U-BLOX NEO-M8N
GPS ^2^ antenna	Taoglass AGGBP.25B.07.0060A
Battery	GEB battery OEM ^3^ 4000 mAh ^4^ Li-Po ^5^ battery
Charger	CUI Inc. SWI10-5-E-I38
Memory flash storage	Winbond Electronics W25Q64FWZPIG8MB
VOC ^9^ sensors	Sensirion SGP30, Bosch BME680

^1^ GSM: Global system for mobile, ^2^ GPS: Global positioning system, ^3^ OEM: original equipment manufacturer, ^4^ mAh: Milliampere hour, ^5^ Li-Po: Lithium polymer, ^6^ IoT: Internet of things, ^7^ M2M: Machine to machine, ^8^ SIM: Subscriber identity module, ^9^ VOC: Volatile organic compounds.

**Table 2:** Overview of the two periods of acquisition; pollutants values in µg/m^3^.

Period	Avg PM_1_^1^	Avg PM_2.5_^1^	Avg PM_10_^1^	Avg NO2 ^2^	Avg BC ^3^	Count PM ^1^	Count NO2 ^2^	Count BC ^3^
A: 10/19/19 to 10/27/19	8.1	11.6	12.9	5.8	0	5325	6040	0
B: 11/14/19 to 11/22/19	8.6	14.5	16.5	7.9	1226.0	4027	4227	2628

^1^ PM: particular matter, ^2^ NO_2_: nitrogen dioxide, ^3^ BC: black carbon.

**Table 3:** Average air pollution level from annotated data, µg/m^3^.

Activity	PM_1.0_^1^	PM_2.5_^1^	PM_10_^1^	NO_2_^2^	BC ^3^
Office	2.3	3.55	3.9	5.7	586.8
Home	10.5	15.1	16.4	6.2	610.0
Shopping	2.7	4.2	4.9	10.4	NA
Park	3.3	4.9	5.3	16.5	409.9
Restaurant	3.6	5.6	6.2	11.2	NA
Street	3.0	4.6	5.1	18.5	NA
Car	9.2	13.1	14.3	20.6	2937.8

^1^ PM: particular matter, ^2^ NO_2_: nitrogen dioxide, ^3^ BC: black carbon.

## Data Availability

The POLLUSCOPE volunteer’s data is made available, however the GPS data has been removed for obvious confidentiality concerns. POLLAR data is not made public as obtaining each patients’ approval isn’t possible.

## Supplementary Materials

The following are available online at https://www.mdpi.com/1424-8220/21/5/1876/s1, Table S1: Individual PM Exposure of the total number of recorded samples.
